# Effect of Structural Uncertainty in Passive Microwave Soil Moisture Retrieval Algorithm

**DOI:** 10.3390/s20041225

**Published:** 2020-02-24

**Authors:** Lanka Karthikeyan, Ming Pan, Dasika Nagesh Kumar, Eric F. Wood

**Affiliations:** 1 Centre of Studies in Resources Engineering, Indian Institute of Technology, Bombay, Powai, Mumbai 400 076, India; 2 Department of Civil Engineering, Indian Institute of Science, Bangalore 560 012, India; dasikanagesh@gmail.com; 3 Department of Civil and Environmental Engineering, Princeton University, Princeton, NJ 08544, USA; mpan@princeton.edu (M.P.); efwood@princeton.edu (E.F.W.)

**Keywords:** soil moisture, equifinality, uncertainty, VOD, passive microwave, retrieval algorithm, AMSR-E, radiative transfer model

## Abstract

Passive microwave sensors use a radiative transfer model (RTM) to retrieve soil moisture (SM) using brightness temperatures (*T_B_*) at low microwave frequencies. Vegetation optical depth (VOD) is a key input to the RTM. Retrieval algorithms can analytically invert the RTM using dual-polarized *T_B_* measurements to retrieve the VOD and SM concurrently. Algorithms in this regard typically use the *τ-ω* types of models, which consist of two third-order polynomial equations and, thus, can have multiple solutions. Through this work, we find that uncertainty occurs due to the structural indeterminacy that is inherent in all *τ-ω* types of models in passive microwave SM retrieval algorithms. In the process, a new analytical solution for concurrent VOD and SM retrieval is presented, along with two widely used existing analytical solutions. All three solutions are applied to a fixed framework of RTM to retrieve VOD and SM on a global scale, using X-band Advanced Microwave Scanning Radiometer-Earth Observing System (AMSR-E) *T_B_* data. Results indicate that, with structural uncertainty, there ensues a noticeable impact on the VOD and SM retrievals. In an era where the sensitivity of retrieval algorithms is still being researched, we believe the structural indeterminacy of RTM identified here would contribute to uncertainty in the soil moisture retrievals.

## 1. Introduction

Passive microwave satellite sensors measure the thermal emissions from the Earth’s surface in the form of brightness temperatures (*T_B_*). At appropriate frequencies (in general, L-, C-, X-bands), these sensors assist in obtaining the soil moisture (SM) on a global scale [1]. The retrieval of SM using passive microwave satellite sensors requires an algorithm, which can be used to convert the *T_B_* into SM. Given that a body naturally emits radiation, a landmass covered with a canopy would release thermal emissions primarily from the soil surface, canopy, atmosphere, and cosmic background. These effects are modeled using a radiative transfer model (RTM), which acts as a core component of the retrieval algorithm. Ideally, a retrieval algorithm should be able to decompose the total *T_B_* measured by a satellite into contributions from the components described above.

In general, a radiative transfer model (RTM) forms a core component of a retrieval algorithm. The premise of an RTM is that the brightness temperatures are directly proportional to the dielectric properties of the soil–water medium. The emissions from surface soil are initially attenuated by the soil roughness, which enhances the emissions and, at the same time, mixes them across the polarizations. These roughness effects are, in general, quantified primarily using two parameters, roughness parameter (*h*), and polarization mixing ratio (*Q*), among others [2,3]. In the lower frequencies (which are sensitive to SM variations), the atmosphere is generally considered to be transparent [4], resulting in the omission of attenuations due to the atmosphere and cosmic background. Apart from these factors, the vegetation attenuation is found to have a significant impact on the observed *T_B_* value [5].

The most widely used RTM is the *τ-ω* vegetation model proposed by [6]. The *τ-ω* model is a zeroth-order RTM, which omits the multiple scattering effects that occur in the canopy structure. The model has two parameters, the vegetation optical depth (VOD) (*τ*) and the single scattering albedo (*ω*). The VOD quantifies the water content in the foliage and woody components of the above-ground biomass [7]. Since emissions from the vegetation are directly influenced by the canopy water content, several works have attempted to derive vegetation information that complements the corresponding information obtained from optical indices such as the normalized difference vegetation index (NDVI), enhanced vegetation index (EVI), etc. [8,9,10]. On the other hand, *ω* determines the scattering effects of the canopy layer, expressed as a ratio of the scattering efficiency to the total extinction efficiency.

VOD is an important parameter required to retrieve SM using the RTM. In general, the VOD is estimated in two ways; one way is to use ancillary data, such as the NDVI, and empirical relationships that estimate the VOD [11]. Such methods can use only a single-channel algorithm (SCA) (such as in the case of the soil moisture active passive (SMAP) SM algorithm, which uses *V-*polarization) to estimate soil moisture. The other way is to use the RTM equations available through either single or multi-angular dual-polarized (such as in the case of the soil moisture ocean salinity (SMOS) SM algorithm) brightness temperature observations and estimate the VOD simultaneously with the SM [12,13,14]. The focus of this study lies in the latter method, i.e., estimating VOD and SM concurrently.

The soil moisture retrievals obtained from microwave observations are prone to uncertainties from various sources. If we consider the input data, the dual-polarized *T_B_* observations (used in the retrieval algorithm) are highly correlated by nature, due to which there can be a certain redundancy in terms of representing the Earth’s surface characteristics between these two observations. As pointed out by [15], this correlated nature can result in the under-determined problem of estimating two unknowns of SM and VOD. This issue leads to compensating errors between SM and VOD, which lead to an observation that the dual-channel algorithms are prone to greater errors in SM than the single-channel algorithm [16,17]. Recently, this problem has been tackled by using *T_B_* observations from multiple overpasses [18,19,20], and by using *T_B_* observations from multiple incidence angles [12,21]. Attempts have also been made to fuse microwave and optical satellite data to estimate SM and VOD concurrently, along with surface roughness information [22].

Further, the RTM also consists of parameters that characterize the surface roughness, and the scattering nature of vegetation. These parameters are necessary to accurately simulate the emissivity from soil and vegetation. For instance, reference [3] calibrated the surface roughness and single scattering albedo parameters by considering a set of candidate parameters and an optimization scheme to improve the quality of SMOS soil moisture retrievals. Although attempts are made to assess the uncertainties due to RTM parameterization [23], to the best of our knowledge, there are no studies that focused on model structural uncertainties on soil moisture retrievals.

In terms of using dual-polarized *T_B_* observations, the *τ-ω* vegetation model in the two polarizations can be used to estimate SM and VOD simultaneously. The equations are, primarily, the nonlinear functions of VOD and SM, along with other variables and parameters, i.e., two third-order polynomial equations of VOD and soil emissivity (a nonlinear function of SM). Given the mathematical nature of these equations, there exists an inherent structural indeterminacy leading to the possibility of the existence of multiple ways by which SM and VOD can be estimated. The study in [13] analytically solved the *τ-ω* model in the context of the land parameter retrieval algorithm (LPRM). Further, reference [14] obtained another unique solution in the context of the land surface microwave emission model (LSMEM). These works attempted to concurrently estimate VOD and SM using a snapshot of dual-polarized *T_B_* observations. In addition, reference [10] proposed a similar solution in the context of the University of Montana (UMT) retrieval algorithm. However, the UMT algorithm also involves the retrieval of additional parameters, such as the water fraction and surface temperature. Although the analytical solutions in these works are intended for the same purpose (for SM estimation), each of their solutions’ structures are mathematically unique, which can result in a unique value of SM retrieval for the same input and parameter configurations of RTM. This model structural indeterminacy can also be called the problem of equifinality in the *τ-ω* model. Equifinality is defined as the possibility of reaching an acceptable model outcome through different model structures or parameterizations within a model framework [24].

With this background, we attempted to address the following objectives through this work: (1) investigate if structural indeterminacy can alter the outcome of soil moisture retrievals and (2) assess the sensitivity of RTM parameterization on the problem of equifinality. To depict the structural indeterminacy, we present a new solution that simultaneously estimates the VOD and SM using dual-polarized *T_B_* information from the RTM scheme. The analysis is carried out using advanced microwave scanning radiometer (AMSR)-E X-band *T_B_* data on a global scale. Currently, attempts are being made to obtain a consistent long record of satellite-based SM retrievals [25,26], by including data from the operational satellite SM sensors soil moisture ocean salinity (SMOS) and soil moisture active passive (SMAP). Evidently, there is a growing need to assess the uncertainty due to the problem of equifinality (structural indeterminacy), which has not been identified before this study. Section 2 presents the RTM scheme and discusses the problem of equifinality. Section 3 presents the data and the experimental setup. Section 4 presents the results and discussion. Section 5 presents the important conclusions drawn from this study.

## 2. RTM Model Setup

The following equations present the zeroth-order *τ-ω* radiative transfer model [6]:

(1) $$T_{BH}=T_{S}\varepsilon_{rH}\Gamma_{C}+T_{C}\left(1-\omega \right)\left(1-\Gamma_{C}\right)+T_{C}\left(1-\omega \right)\left(1-\Gamma_{C}\right)\left(1-\varepsilon_{rH}\right)\Gamma_{C}$$

(2) $$T_{BV}=T_{S}\varepsilon_{rV}\Gamma_{C}+T_{C}\left(1-\omega \right)\left(1-\Gamma_{C}\right)+T_{C}\left(1-\omega \right)\left(1-\Gamma_{C}\right)\left(1-\varepsilon_{rV}\right)\Gamma_{C}$$

where, *H* and *V* indicate the horizontal and vertical polarizations, respectively, $T_{S}$ and $T_{C}$ are the soil and canopy temperatures, respectively, $\varepsilon_{r}$ is the emissivity of a rough soil surface, and $\Gamma_{C}$ is the vegetation transmissivity. These equations simulate the brightness temperatures of (1) direct emission from the soil, (2) upward emission from the canopy, and (3) ground-reflected downward emission from the canopy. The vegetation transmissivity is obtained from the following equation.

(3) $$\Gamma_{C}=\exp\left(-\tau \sec\theta \right)$$

where, τ is the VOD, and θ is the angle of incidence. The emissivity of a rough soil is estimated from the smooth soil emissivity upon accounting for the soil roughness effects using the *h–Q* model [2,27]. *h* and *Q*, which are the roughness parameter and the polarization mixing ratio, respectively, are computed using the following equations.

(4) $$h=4h_{rms}^{2}{\left(2\pi f/\left(c\times 100\right)\right)}^{2}$$

(5) $$Q=0.35\times \left(1-\exp\left(-0.6\cdot h_{rms}\cdot f\cdot 10^{-9}\right)\right)$$

where, $h_{rms}$ is the root mean square height, which is assumed to be a global constant value of 0.3 cm [27]; *f* is the frequency of channel in Hz; c is the speed of light ($=3\times 10^{8}\;m/s$). Upon calculating values of *h* and *Q*, the rough soil emissivity is estimated using:

(6) $$\begin{array}{l} \varepsilon_{rH}=1-\left[\left(1-Q\right)\left(1-\varepsilon_{sH}\right)+Q\left(1-\varepsilon_{sV}\right)\right]\exp\left(-h\cos^{n}\theta \right)\\ \varepsilon_{rV}=1-\left[\left(1-Q\right)\left(1-\varepsilon_{sV}\right)+Q\left(1-\varepsilon_{sH}\right)\right]\exp\left(-h\cos^{n}\theta \right) \end{array}$$

where, $\varepsilon_{sV}$ and $\varepsilon_{sH}$ are the vertically and horizontally polarized smooth soil emissivities, respectively, *n* is assigned a constant global value of 2 [2]. The smooth soil emissivity is estimated primarily from the soil dielectric constant (using Fresnel equations, [28]), which in turn is estimated from SM content using a dielectric mixing model. Further information on the retrieval algorithms and the associated developments can be obtained from [28,29]. The RTM equations are inverted analytically using a new procedure, which is presented below, to retrieve VOD and SM. The inversion involves the following assumptions, (1) $T_{S}\approx T_{C}$, (2) *ω* is polarization independent and is assigned a global constant value of 0.07 [30,31], (3) $\Gamma_{C}$ is polarization independent, and (4) the roughness parameter (*h*) used in the h−Q model is polarization independent.

Under the above model conditions, the following equations present the $\Gamma_{C}$ solution proposed by [13,14], which are termed as $\Gamma_{C,Pan}$ and $\Gamma_{C,Meesters}$, respectively.

(7) $$\Gamma_{C,Pan}=\frac{1}{2\left(1-\omega \right)}\left[\sqrt{\omega^{2}+\frac{4\left(1-\omega \right)\left(T_{BV}-T_{BH}\right)}{T_{S}\left(\varepsilon_{rV}-\varepsilon_{rH}\right)}}-\omega \right]$$

(8) $$\frac{1}{\Gamma_{C,Meesters}}=ad+\sqrt{{\left(ad\right)}^{2}+a+1},\mathrm{where}\;\begin{array}{ll} a & =\frac{1}{2}\left\{\frac{\varepsilon_{rV}-\varepsilon_{rH}}{MPDI}-\left(\varepsilon_{rV}+\varepsilon_{rH}\right)\right\}\\ & d=\frac{1}{2}\frac{\omega }{1-\omega } \end{array}$$

where, *MPDI* is the microwave polarization difference index, which is calculated as $MPDI=\left(T_{BV}-T_{BH}\right)/\left(T_{BV}+T_{BH}\right)$. 

### Analytical Derivation—New Solution

Under the above model setup, adding and subtracting Equations (1) and (2) results in the following equations.

(9) $$T_{BH}+T_{BV}=T_{S}\Gamma_{C}\left(\varepsilon_{rH}+\varepsilon_{rV}\right)\left(1-\left(1-\omega \right)\left(1-\Gamma_{C}\right)\right)+2T_{S}\left(1-\omega \right)\left(1-\Gamma_{C}^{2}\right)$$

(10) $$T_{BH}-T_{BV}=T_{S}\Gamma_{C}\left(\varepsilon_{rH}-\varepsilon_{rV}\right)\left(1-\left(1-\omega \right)\left(1-\Gamma_{C}\right)\right)$$

Equation (10) can be rewritten in the following form.

(11) $$\left(1-\left(1-\omega \right)\left(1-\Gamma_{C}\right)\right)=\frac{T_{BH}-T_{BV}}{T_{S}\Gamma_{C}\left(\varepsilon_{rH}-\varepsilon_{rV}\right)}$$

Substituting Equation (11) in Equation (9) results in

(12) $$T_{BH}+T_{BV}=\left(\varepsilon_{rH}+\varepsilon_{rV}\right)\frac{T_{BH}-T_{BV}}{\left(\varepsilon_{rH}-\varepsilon_{rV}\right)}+2T_{S}\left(1-\omega \right)\left(1-\Gamma_{C}^{2}\right)$$

Simplifying the above final equation results in the new analytical solution of $\Gamma_{C}$ termed as $\Gamma_{C,New}$. The complete derivation for $\Gamma_{C,New}$ is provided in Supplementary Material S1 (Figure S1).

(13) $$\Gamma_{C,New}=\sqrt{\frac{\left(\varepsilon_{rH}T_{BV}-\varepsilon_{rV}T_{BH}\right)}{T_{S}\left(1-\omega \right)\left(\varepsilon_{rV}-\varepsilon_{rH}\right)}+1}$$

The following iterative procedure describes the manner by which the SM is retrieved using $\Gamma_{C}$. (1) Start with an initial guess of SM and estimate soil dielectric constant using Dobson dielectric mixing model [32]. (2) Estimate the smooth soil emissivity in horizontal and vertical polarizations, $\varepsilon_{sH}$ and $\varepsilon_{sV}$, respectively, using Fresnel’s equations [28]. (3) Estimate the rough soil emissivity in horizontal and vertical polarizations, $\varepsilon_{rH}$ and $\varepsilon_{rV}$, respectively, using roughness equations (Equation (6)). (4) Estimate $\Gamma_{C}$ by substituting $\varepsilon_{rH}$ and $\varepsilon_{rV}$, along with $T_{BH}$, $T_{BV}$, $T_{S}$ and ω, in either Equation (7) or Equation (8) or Equation (13). It may be noted that satellite observed brightness temperature values are used in these equations to compute $\Gamma_{C}$. VOD (τ) can be estimated from $\Gamma_{C}$ by inverting Equation (3). (5) Simulate $T_{BH}$ and $T_{BV}$ using Equations (1) and (2), respectively with $\Gamma_{C}$, $\varepsilon_{rH}$, $\varepsilon_{rV}$, $T_{S}$ and ω as inputs. (6) Calculate SM by minimizing the root mean square between observed and simulated $T_{BH}$ and $T_{BV}$, using bisection algorithm. (7) Repeat steps two to six until the error value is at the minimum or the iterations converge. The outcomes of the SM and VOD (computed from $\Gamma_{C}$) obtained at the end of iterations are considered the final retrievals that correspond to the observed $T_{BH}$ and $T_{BV}$ for an overpass.

**Figure 1:**
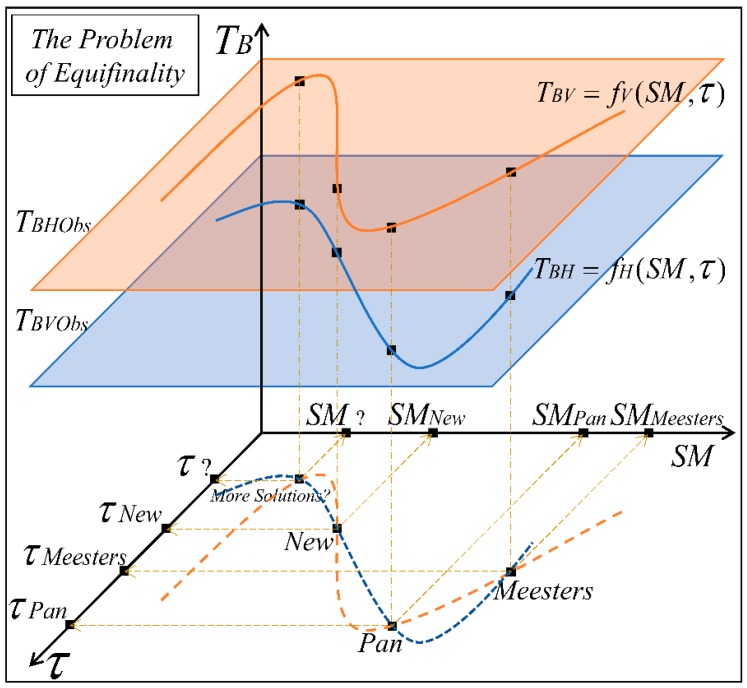
The problem of equifinality (structural indeterminacy) in the concurrent retrieval of soil moisture (SM) and vegetation optical depth (*τ*). The three axes represent brightness temperatures (*T_B_*), SM and *τ*. The two planes shaded in orange and blue correspond to observed satellite measurements *T_BVObs_* and *T_BHObs_* at a timestep, respectively (in *V* and *H* polarizations, respectively). The thick curves shown in orange and blue on these planes represent the RTM equations in *V* and *H*, respectively (Equations (1) and (2)), which are primarily the functions of SM and *τ*. The dashed lines on the SM–τ plane are the projections of the solid curves. The points where these dashed lines intersect (black squares) on the SM–τ plane are the possible solutions to both RTM equations. Using the Pan solution results in (SM*_Pan_*, *τ_Pan_*), the New solution results in (SM*_New_*, *τ_New_*), and the Meesters solution results in (SM*_Meesters_*, *τ_Meesters_*). The problem of equifinality arises with the fact that all three solutions lead to the similar *T_BV_* and *T_BH_* values. The existence of more solution(s) is also possible (‘More Solutions?’ in the SM–τ plane).

The prime difference among the solutions lies in the fact that, while the Pan and Meesters solutions solve the positive roots of the quadratic equations of $\Gamma_{C}$ to result in $\Gamma_{C,Pan}$ and $\Gamma_{C,Meesters}$, respectively, the new solution involves a pure quadratic equation of $\Gamma_{C}$ (Equation (13); with no linear term of $\Gamma_{C}$), which is directly solved to result in $\Gamma_{C,New}$. It can be noticed from Equations (7), (8) and (13) that there exist multiple ways by which $\Gamma_{C}$ can be derived by solving the RTM equations (Equations (1) and (2)). As the formulation of $\Gamma_{C}$ varies according to the type of solution, the VOD varies, which ultimately alters the SM retrieval. So, given a set of dual-polarized satellite measures $T_{B}$ on a particular day, there are multiple sets of optimized retrieval pairs of VOD and SM that exist in their respective physical ranges. This structural indeterminacy, which translates to the problem of equifinality in the SM retrieval algorithm, is illustrated in Figure 1. With this finding, we speculate that there is also a possibility of the existence of some more solutions to this problem. In this regard, attempts are made to solve the equations by deriving from them a fourth-order polynomial of $\Gamma_{C}$, from which four solutions could be derived. After discarding the two negative roots, the two positive roots are initially thought of as plausible solutions. However, they are found to be unstable as the iterative procedure does not converge.

## 3. Data and Application

Since the formulation of RTM equations is independent of microwave frequency and sensors, the associated $\Gamma_{C}$ solutions apply to any satellite sensor, which has the SM sensitive frequencies onboard. In order to assess the problem of equifinality caused by the three $\Gamma_{C}$ solutions, in this letter, AMSR-E X-band (10.65 GHz) frequency Level 3 $T_{B}$ data are used to retrieve SM at the global scale. The $T_{B}$ used is of Level 3 type (version 3) [33], having a grid resolution of 0.25° × 0.25°, available at daily scale for two passes (ascending and descending) with an angle of incidence of 55°. The data are available from 19 June 2002 to 27 September 2011. The surface temperature ($T_{S}$), an input required in the retrieval process, is estimated from the linear regression relationships developed by [34] that take vertical polarization Ka-band (36.5 GHz) $T_{B}$ data as the input ($T_{BVKa}$). The following equations are used for computing $T_{S}$ for the two passes.

(14) $$\begin{array}{l} T_{S}=0.898\times T_{BVKa}+44.2\to \mathrm{Ascending}\;\mathrm{Pass}\\ T_{S}=0.893\times T_{BVKa}+44.8\to \mathrm{Descending}\;\mathrm{Pass} \end{array}$$

The RTM equations presented here correspond to the emissions from a landmass. So, it is necessary that the total $T_{B}$ observed at a grid location should be decomposed into the individual contributions from land and water in the following way:

(15) $$T_{BObs}=f_{Land}\cdot T_{BLand}+f_{Water}\cdot T_{BWater}.$$

The fraction of vegetation (translated as the land here; in the case of bare soil, the vegetation properties would be nullified) is obtained from moderate resolution imaging spectroradiometer (MODIS) vegetation index data product MOD12Q1 [35,36]. This parameter varied according to monthly climatology. On the other hand, the fraction of water ($f_{Water}$) is considered to be a static parameter, which is obtained from MODIS land cover classification product MOD13A2 [37]. $T_{BWater}$ is estimated using the equations of bare soil RTM (consisting of the only first term in Equations (1) and (2)) where the rough soil emissivity ($\varepsilon_{r}$) is replaced with that of water, and the soil temperature is replaced with water temperature. The open water emissivity values of 0.5791 (*V*-pol) and 0.2827 (*H*-pol) are assumed. It is reasonable to assume constant values of open water emissivities for inland water bodies at low microwave frequencies (<18 GHz) [10]. Once $f_{Land}$, $f_{Water}$, and $T_{BWater}$ are known, $T_{BLand}$ can be estimated based on $T_{BObs}$ by inverting Equation (15). The conversion of SM to soil dielectric constant is carried out using the Dobson model [32]. SM of the AMSR-E X-band $T_{B}$ data are retrieved at the global scale through the three analytical solutions (thus forming three SM products) using the RTM framework mentioned above. Since the RTM scheme remains constant across the three SM products, all the attributable changes in the SM correspond to the change in analytical solution.

## 4. Results and Discussion

**Figure 2:**
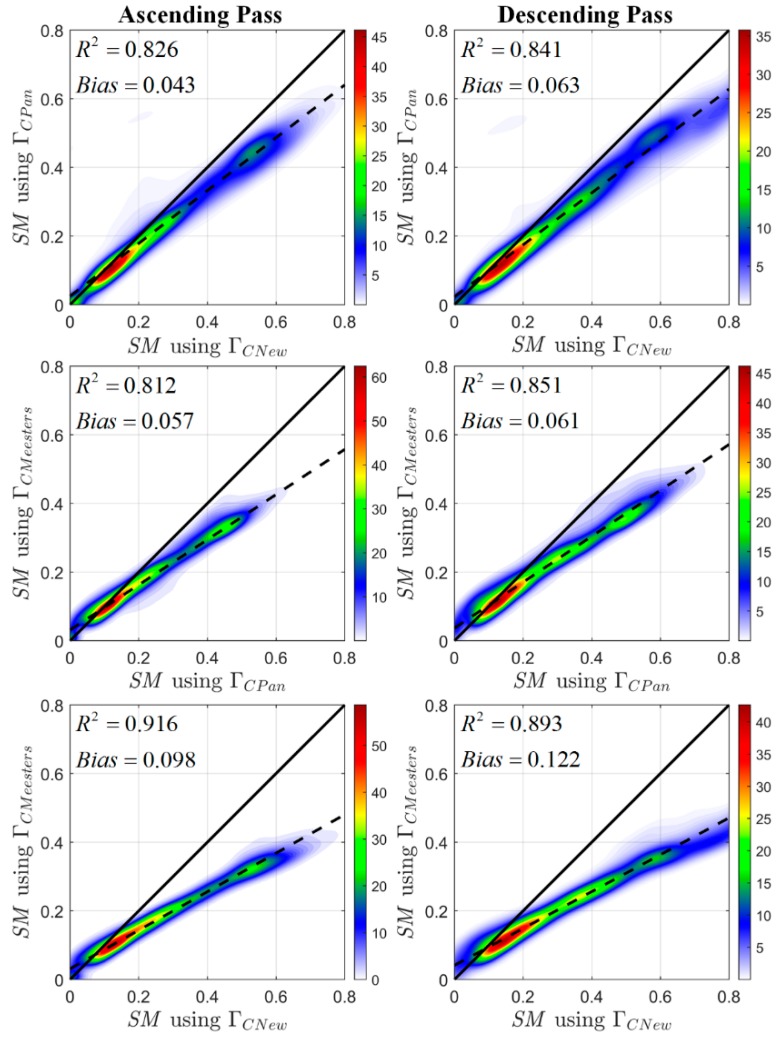
Scatter density plots of mean advanced microwave scanning radiometer (AMSR)-E SM of the retrievals obtained by employing three analytical solutions in the RTM framework. Retrievals corresponding to ascending and descending passes are plotted in the first and second columns of the figure, respectively. The thick and the dotted lines in each plot represent the normal line (45° line) and best fit line, respectively.

Figure 2 presents the scatter density plots between temporal mean SM values of the three products computed at all land locations of the globe. The numbers in each plot indicate the spatial mean of the temporal coefficient of determination ($R^{2}$) and the spatial mean of temporal bias (estimated as $E[SM_{X-axis}]-E[SM_{Y-axis}]$, expressed in $m^{3}/m^{3}$). The plots depict that $\Gamma_{C,New}$ has a tendency to overestimate the SM compared to that of the other two solutions. This overestimation is prominent for the wetter soils. Relatively higher VOD retrievals are obtained through the usage of $\Gamma_{C,New}$ compared to the other two solutions (results presented in Supplementary Material S2), resulting in low transmissivity values (Equation (3)), which means that this solution models vegetation much more thickly than the other two solutions. Low values of transmissivity can lead to colder estimates of $T_{B}$, which ultimately results in higher values of retrieved SM (much wetter soils). The SM from $\Gamma_{C,Meesters}$ is found to be lowest in terms of magnitude compared to the other two products. In terms of $R^{2}$, values as low as 0.812 (ascending pass, between SM obtained from $\Gamma_{C,Pan}$ and $\Gamma_{C,Meesters}$) and 0.841 (descending pass, between SM obtained from $\Gamma_{C,New}$ and $\Gamma_{C,Pan}$) are obtained, which indicate that, approximately, between 19% and 16% of unexplained variances, respectively, are caused due to changes in the analytical vegetation transmissivity solution. In the case of the spatial mean of temporal bias, a positive bias of 0.098 $m^{3}/m^{3}$ and 0.122 $m^{3}/m^{3}$ are obtained for ascending and descending passes, respectively, between SM retrieved using $\Gamma_{C,Pan}$ and $\Gamma_{C,Meesters}$. These numbers indicate that there is a significant effect of equifinality on the SM retrievals when simultaneously estimating VOD and SM in the passive microwave SM retrieval algorithm.

**Figure 3:**
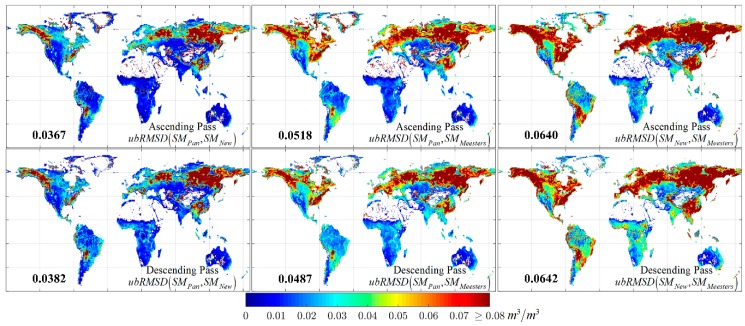
Unbiased root mean squared difference (ubRMSD) between the SM products obtained using the three solutions $\Gamma_{C,New}$ (indicated as SM*_New_*), $\Gamma_{C,Pan}$ (indicated as SM*_Pan_*) and $\Gamma_{C,Meesters}$ (indicated as SM*_Meesters_*) for ascending and descending passes. The number in bold in each tile indicate the global average of ubRMSD computed between corresponding SM products.

The uncertainty in SM retrievals caused due to structural indeterminacy is assessed further by computing the unbiased root mean squared difference (ubRMSD) between the three SM products for ascending and descending passes. Figure 3 presents the maps of ubRMSD. Each of these maps also contain the global average of ubRMSD computed between corresponding SM products. Results indicate that the errors between SM retrieved using $\Gamma_{C,New}$ and $\Gamma_{C,Meesters}$ are highest followed by the errors between SM retrieved using $\Gamma_{C,Pan}$ and $\Gamma_{C,Meesters}$. High uncertainty exists among the retrievals in the boreal landscapes in higher latitudes, portions of Southeast Asia, Bolivia, and Eastern USA. Since these regions are dominated by vegetation cover, the effects of alteration between the three solutions of $\Gamma_{C}$ are much more prominent. Noticeable uncertainties are also found in the croplands of Brazil and Southern Africa, the tropical savanna region between the Sahara Desert and the Congo basin in Africa and Central India. The global average of ubRMSD indicates that the errors range from 0.0367 to 0.0642 $m^{3}/m^{3}$. Since these results now depict that equifinality can be one of the contributing factors to the total uncertainty in passive microwave SM retrievals (which are retrieved along with VOD), we speculate that these errors could have contributed towards the total error obtained by validating the AMSR-E LSMEM and AMSR-E LPRM SM products over the Contiguous United States (CONUS) region using the in situ observations [38]. 

We carried out a sensitivity analysis to assess the extent of the parameterization of the retrieval algorithm on the retrievals of VOD and SM. In this experiment, eight locations are randomly selected (Table S1) across the globe and considered against the effects of three parameters *h*, *Q*, and *ω*. The ranges of these three parameters are presented in Table 1. We obtained 50,000 unique combinations of the three-parameter sets using Latin hypercube sampling (LHS). We retrieved VOD and SM pertaining to these parameter sets across the eight sites for fixed values of $T_{BH}$, $T_{BV}$, $T_{BVKa}$ (which is used to compute the surface temperature $T_{S}$) corresponding to June 21, 2002 (presented in Table S2). 

**Figure 4:**
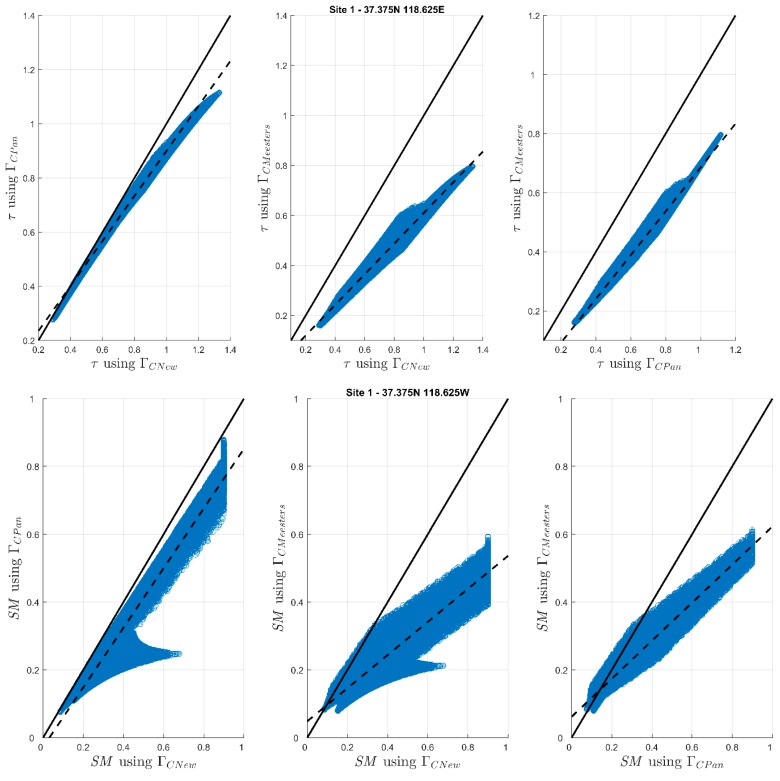
Scatter plots of VOD (top row) and SM (bottom row) simulations pertaining to 50,000 parameter sets (*h*, *Q*, *ω*) obtained by altering the analytical solution at Site 1.

Figure 4 presents the scatter plots of VOD and SM retrievals, pertaining to the 50,000 parameter sets, obtained by altering the analytical solutions at Site 1. Scatter plots for the rest of the sites are presented in Supplementary Material S3 (Figures S2–S8). Results from the experiment indicated that the three solutions retrieved the VOD and SM with noticeable differences, irrespective of the changes in the parameterization of the retrieval algorithm. This further corroborates our hypothesis that the structural indeterminacy (equifinality) in the passive microwave soil moisture retrieval algorithm indeed has a significant impact on the VOD and SM retrievals. 

## 5. Conclusions

The objective of this work is to present the problem of structural indeterminacy (equifinality) in the passive microwave soil moisture retrieval algorithm and showcase the extent to which an analytical solution can affect the SM retrievals. In this process, a new analytical solution is presented to derive vegetation transmissivity, which is estimated, along with SM, using the zeroth-order radiative transfer scheme. This solution, along with two of the existing solutions (proposed by [14] and [13]), is used in a fixed framework of RTM to retrieve SM from AMSR-E $T_{B}$ data. Results indicated the three soil moisture products to have noticeable differences in terms of bias, unexplained variance (through $R^{2}$), and ubRMSD. Equifinality is found to persist independent of the RTM parameterizations. 

It is necessary to note that validating these soil moisture products may not be straightforward. This is because there cannot be “one” solution that can outperform the other two solutions in terms of soil moisture retrievals, irrespective of land cover or meteorological or soil conditions. As a result, attributing the performance of the soil moisture product to the mathematical structure of the $\Gamma_{C}$ solution embedded in RTM is a challenging task. Since attempts are now being made to apply a retrieval algorithm to new sensor data (for example, applying the LPRM to advanced microwave scanning radiometer 2 (AMSR2) data [39] and to SMOS data [40]), it would be interesting to interpret the problem of structural indeterminacy on AMSR2, SMOS and SMAP datasets. In light of these new findings, there is a need to rethink the tradeoff between model complexity/physicality versus the retrieval uncertainties, which aid a comprehensive evaluation of the quality of resulting SM retrievals in the future.

## Figures and Tables

**Table 1:** Parameters and their ranges considered for the sensitivity analysis.

Parameter	Range
*h*	0–3.2
*Q*	0–0.2
*ω*	0–0.1

## Supplementary Materials

The Supplementary Materials are available online at https://www.mdpi.com/1424-8220/20/4/1225/s1.
