# Supplementary material for: Effect of Structural Uncertainty in Passive Microwave Soil Moisture Retrieval Algorithm
Source: Sensors (Basel). 2020 Feb 24;20(4):1225. doi: 10.3390/s20041225 (PMC7070557; doi:10.3390/s20041225)
Supplement: Supplementary file 1 [file sensors-20-01225-s001.pdf]

sensors

Supplementary Material for Effect of Structural Uncertainty in Passive Microwave Soil Moisture Retrieval Algorithm

S1 Complete derivation of $\Gamma_{C,New}$

$$\begin{aligned} T_{BH} &= T_S \varepsilon_{rH} \Gamma_C + T_C (1-\omega)(1-\Gamma_C) + T_C (1-\omega)(1-\Gamma_C)(1-\varepsilon_{rH})\Gamma_C \\ T_{BV} &= T_S \varepsilon_{rV} \Gamma_C + T_C (1-\omega)(1-\Gamma_C) + T_C (1-\omega)(1-\Gamma_C)(1-\varepsilon_{rV})\Gamma_C \end{aligned} \tag{1}$$

Assuming $T_S \approx T_C$

$$\begin{aligned} T_{BH} &= T_S \varepsilon_{rH} \Gamma_C + T_S (1-\omega)(1-\Gamma_C) + T_S (1-\omega)(1-\Gamma_C)(1-\varepsilon_{rH})\Gamma_C \\ T_{BV} &= T_S \varepsilon_{rV} \Gamma_C + T_S (1-\omega)(1-\Gamma_C) + T_S (1-\omega)(1-\Gamma_C)(1-\varepsilon_{rV})\Gamma_C \end{aligned} \tag{2}$$

Adding T_{BH} and T_{BV} equations (Eq. (2))

$$\begin{aligned} T_{BH} + T_{BV} &= T_S \Gamma_C (\varepsilon_{rH} + \varepsilon_{rV}) + 2T_S (1-\omega)(1-\Gamma_C) + T_S (1-\omega)(1-\Gamma_C)\Gamma_C (2 - \varepsilon_{rH} - \varepsilon_{rV}) \\ &= T_S \Gamma_C (\varepsilon_{rH} + \varepsilon_{rV}) + 2T_S (1-\omega)(1-\Gamma_C) \\ &\quad + 2T_S (1-\omega)(1-\Gamma_C)\Gamma_C - T_S (1-\omega)(1-\Gamma_C)\Gamma_C (\varepsilon_{rH} + \varepsilon_{rV}) \\ &= T_S \Gamma_C (\varepsilon_{rH} + \varepsilon_{rV})\left(1 - (1-\omega)(1-\Gamma_C)\right) + 2T_S (1-\omega)(1-\Gamma_C^2) \end{aligned} \tag{3}$$

Subtracting T_{BH} and T_{BV} equations (Eq. (2))

$$\begin{aligned} T_{BH} - T_{BV} &= T_S \Gamma_C (\varepsilon_{rH} - \varepsilon_{rV}) + T_S (1-\omega)(1-\Gamma_C)(1-\varepsilon_{rH})\Gamma_C - T_S (1-\omega)(1-\Gamma_C)(1-\varepsilon_{rV})\Gamma_C \\ &= T_S \Gamma_C (\varepsilon_{rH} - \varepsilon_{rV}) - T_S (1-\omega)(1-\Gamma_C)\Gamma_C (\varepsilon_{rH} - \varepsilon_{rV}) \\ &= T_S \Gamma_C (\varepsilon_{rH} - \varepsilon_{rV})\left(1 - (1-\omega)(1-\Gamma_C)\right) \end{aligned} \tag{4}$$

$$\Rightarrow \left(1 - (1-\omega)(1-\Gamma_C)\right) = \frac{T_{BH} - T_{BV}}{T_S \Gamma_C (\varepsilon_{rH} - \varepsilon_{rV})} \tag{5}$$

Substitute Eq. (5) in Eq. (3)

$$T_{BH} + T_{BV} = \cancel{T_S} \cancel{\Gamma_C} (\varepsilon_{rH} + \varepsilon_{rV}) \frac{T_{BH} - T_{BV}}{\cancel{T_S} \cancel{\Gamma_C} (\varepsilon_{rH} - \varepsilon_{rV})} + 2T_S (1-\omega)(1-\Gamma_C^2) \tag{6}$$

$$2T_S (1-\omega)(1-\Gamma_C^2) = (T_{BH} + T_{BV}) - (\varepsilon_{rH} + \varepsilon_{rV}) \frac{T_{BH} - T_{BV}}{(\varepsilon_{rH} - \varepsilon_{rV})} \tag{7}$$

$$2T_S (1-\omega)(1-\Gamma_C^2) = \frac{\cancel{T_{BH}\varepsilon_{rH}} - T_{BH}\varepsilon_{rV} + T_{BV}\varepsilon_{rH} - \cancel{T_{BV}\varepsilon_{rV}} - \cancel{T_{BH}\varepsilon_{rH}} + T_{BV}\varepsilon_{rH} - T_{BH}\varepsilon_{rV} + \cancel{T_{BV}\varepsilon_{rV}}}{(\varepsilon_{rH} - \varepsilon_{rV})} \tag{8}$$

$$\cancel{2} T_S (1-\omega)(1-\Gamma_C^2) = \frac{\cancel{2}(\varepsilon_{rH} T_{BV} - \varepsilon_{rV} T_{BH})}{(\varepsilon_{rH} - \varepsilon_{rV})} \tag{9}$$

$$(1-\Gamma_C^2) = \frac{(\varepsilon_{rH} T_{BV} - \varepsilon_{rV} T_{BH})}{T_S (1-\omega)(\varepsilon_{rH} - \varepsilon_{rV})} \tag{10}$$

$$(\Gamma_C^2 - 1) = \frac{(\varepsilon_{rH} T_{BV} - \varepsilon_{rV} T_{BH})}{T_S (1-\omega)(\varepsilon_{rV} - \varepsilon_{rH})} \tag{11}$$

$$\Gamma_C^2 = 1 + \frac{(\varepsilon_{rH} T_{BV} - \varepsilon_{rV} T_{BH})}{T_S (1-\omega)(\varepsilon_{rV} - \varepsilon_{rH})} \tag{12}$$

$$\therefore \Gamma_{C,New} = \sqrt{\frac{(\varepsilon_{rH} T_{BV} - \varepsilon_{rV} T_{BH})}{T_S (1-\omega)(\varepsilon_{rV} - \varepsilon_{rH})} + 1} \tag{13}$$

S2 Scatter density plots of VOD obtained using the three analytical solutions $\Gamma_{C,\,New}$, $\Gamma_{C,\,Pan}$ and $\Gamma_{C,\,Meesters}$

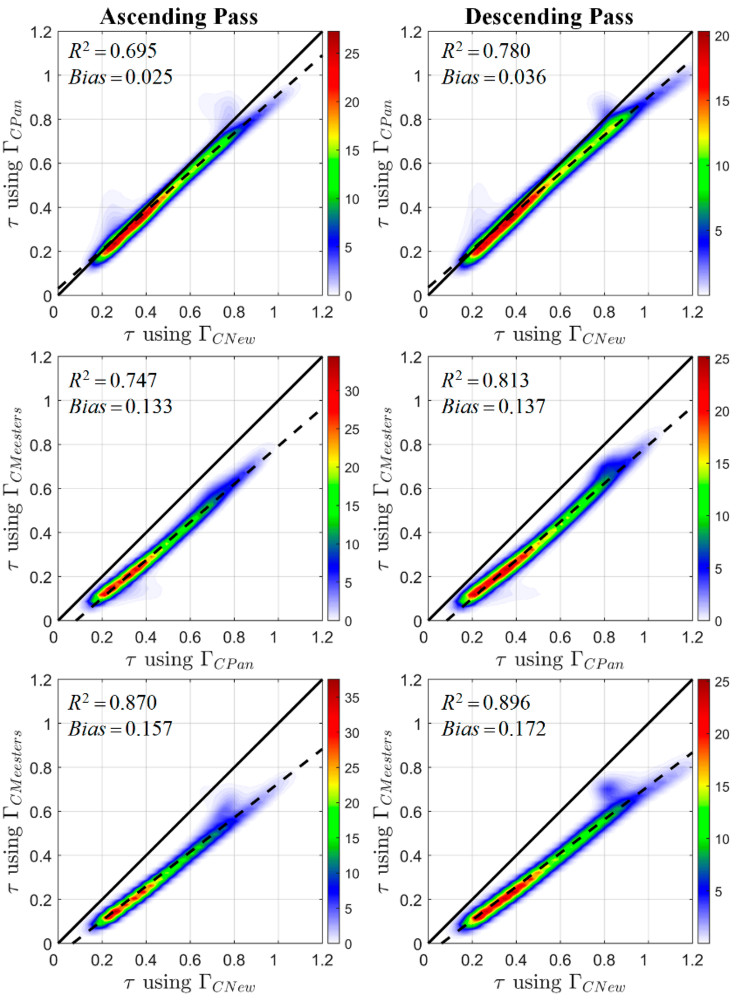

Figure S1. Scatter density plots of mean AMSR-E VOD of the retrievals obtained by employing three analytical solutions in the RTM framework. Retrievals corresponding to ascending and descending passes are plotted in the first and second columns of the figure respectively. The thick and the dotted lines in each plot represent the normal line (45° line) and best fit line respectively.

S3 Sensitivity analysis to assess the extent of parameterization on the problem of equifinality in passive microwave soil moisture retrieval algorithm

Table S1. Details of locations where sensitivity analysis is carried out.

Site	Latitude	Longitude
1	37.375	-118.625
2	40.625	-96.875
3	-35.625	148.125
4	38.875	-120.125
5	31.625	-100.625
6	36.125	-105.625

7	45.375	-75.625
8	38.625	-119.625

Table S2. Values of T_{BH} , T_{BV} , T_{BV36} (in Kelvin) considered across the eight sites for the sensitivity analysis.

Site	T_{BH} (K)	T_{BV} (K)	T_{BV36} (K)
1	256.7	270.2	272.2
2	272.6	282.8	282.5
3	251.8	264.3	266.9
4	261.4	270.0	269.2
5	259.6	270.0	267.4
6	270.0	275.2	275.4
7	267.8	286.2	285.6
8	263.7	269.2	266.8

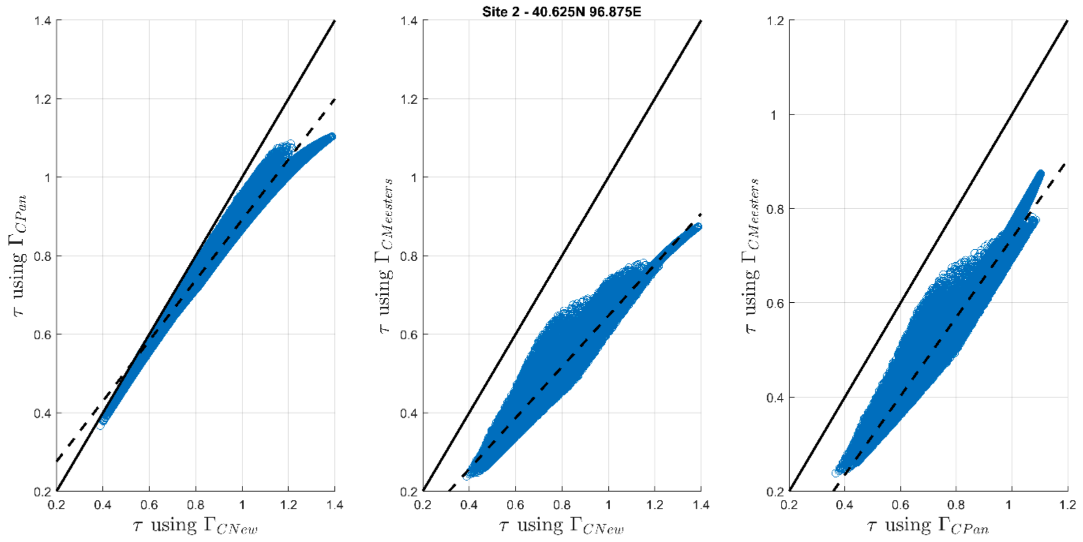

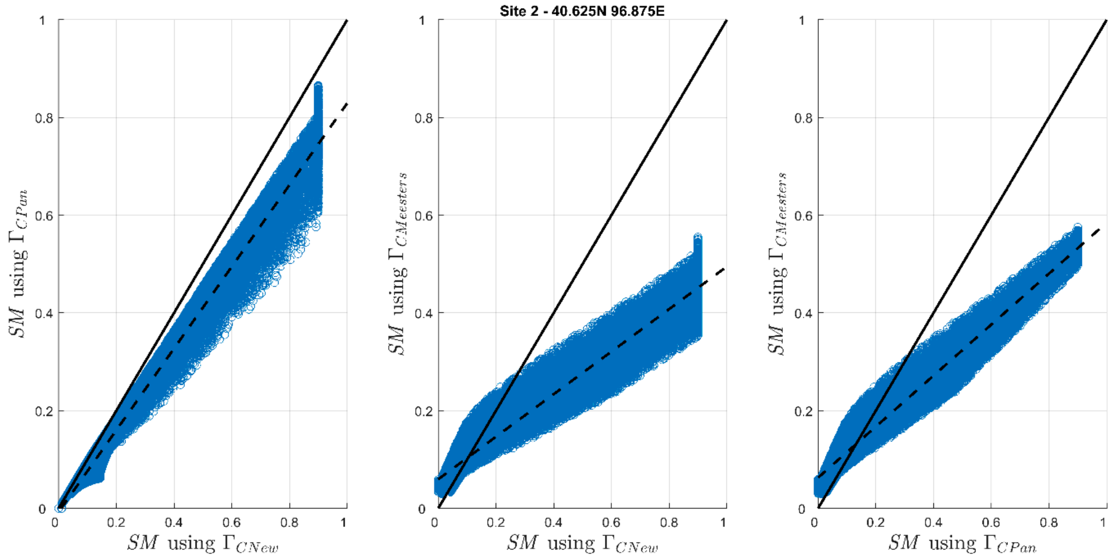

Figure S2. Scatter plots of VOD (top row) and *SM* (bottom row) simulations pertaining to 50000 parameter sets (h, Q, ω) obtained by altering the analytical solution at Site 2.

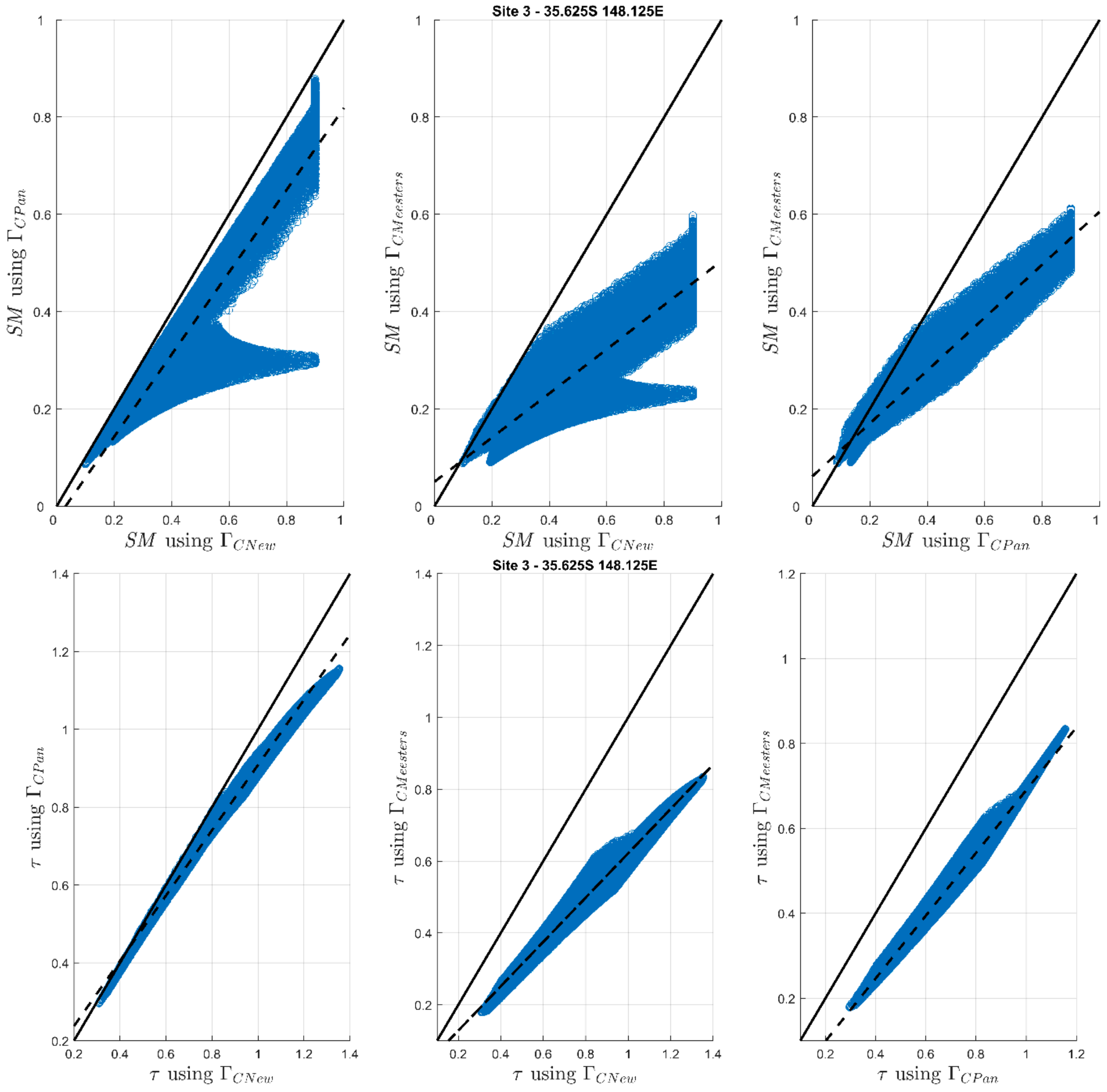

Figure S3. Same as Figure S2, but for Site 3.

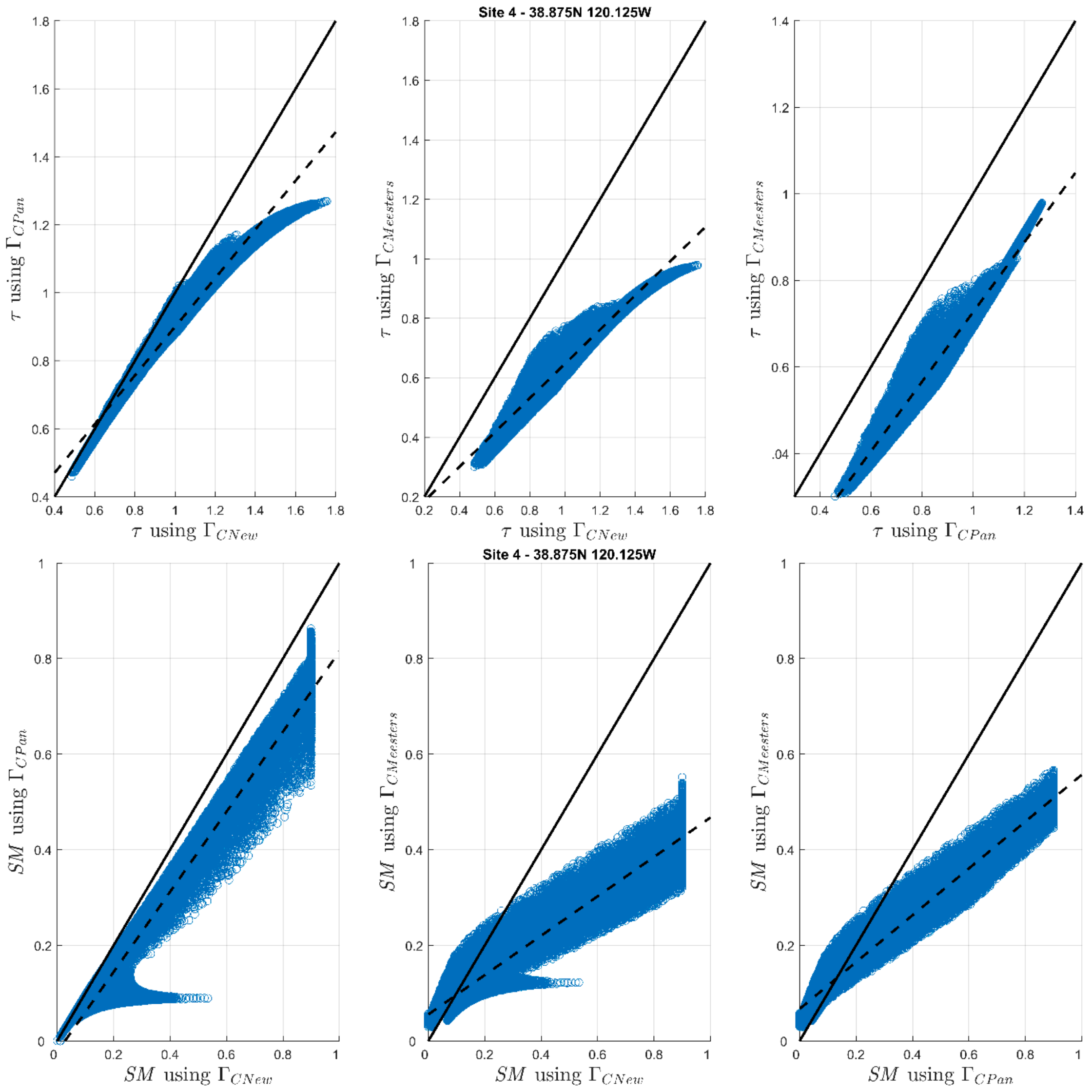

Figure S4. Same as Figure S2, but for Site 4.

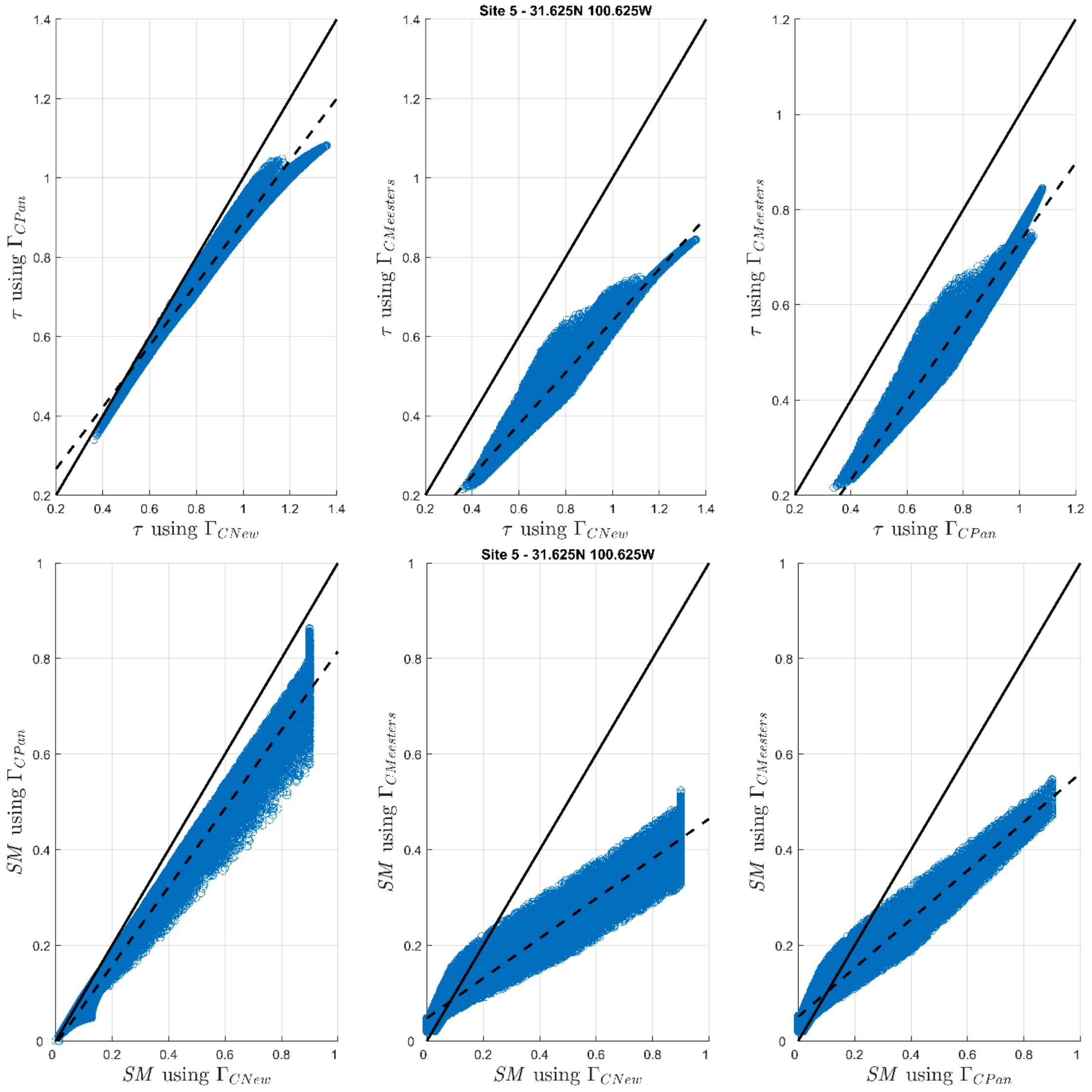

Figure S5. Same as Figure S2, but for Site 5.

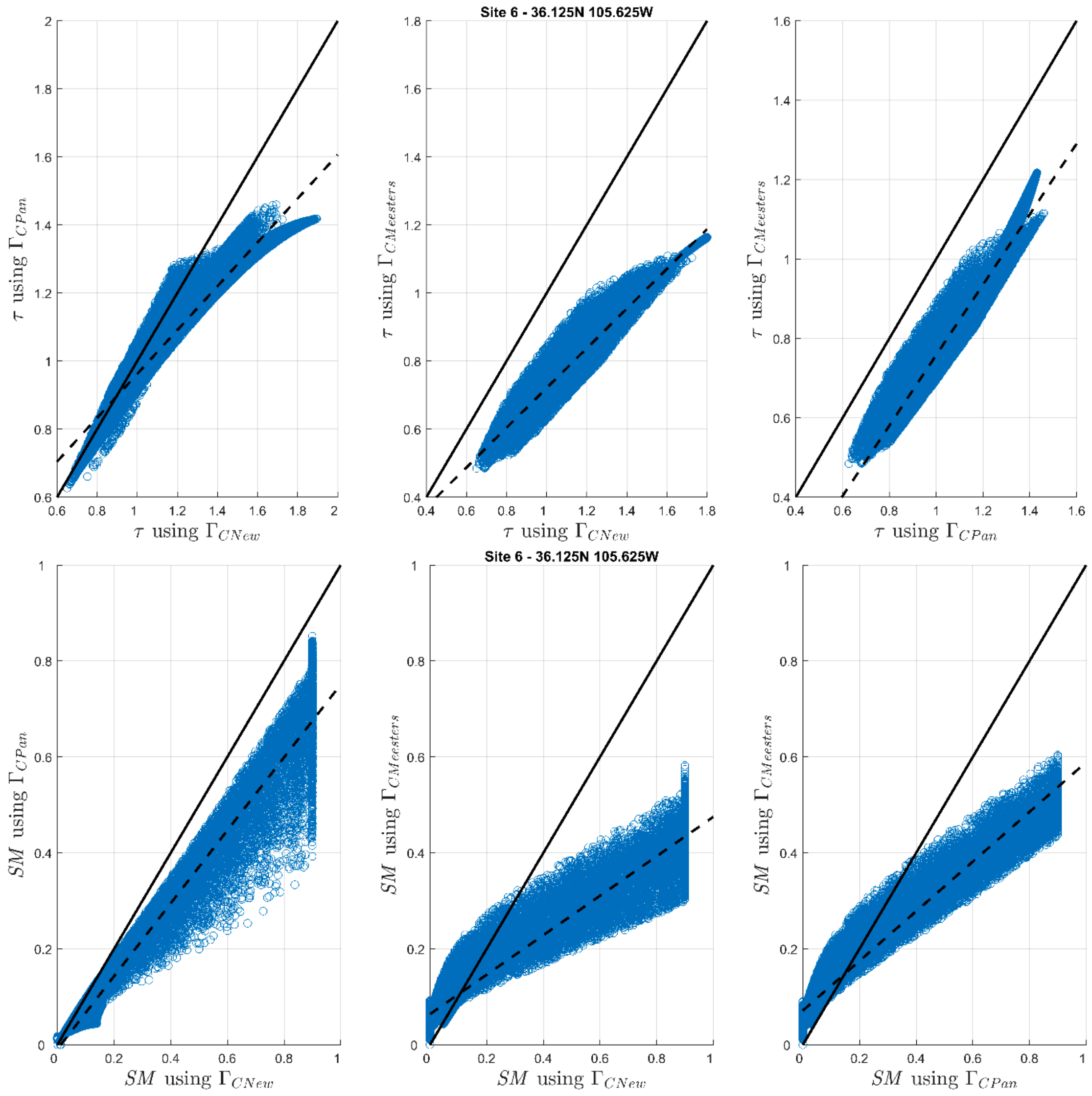

Figure S6. Same as Figure S2, but for Site 6.

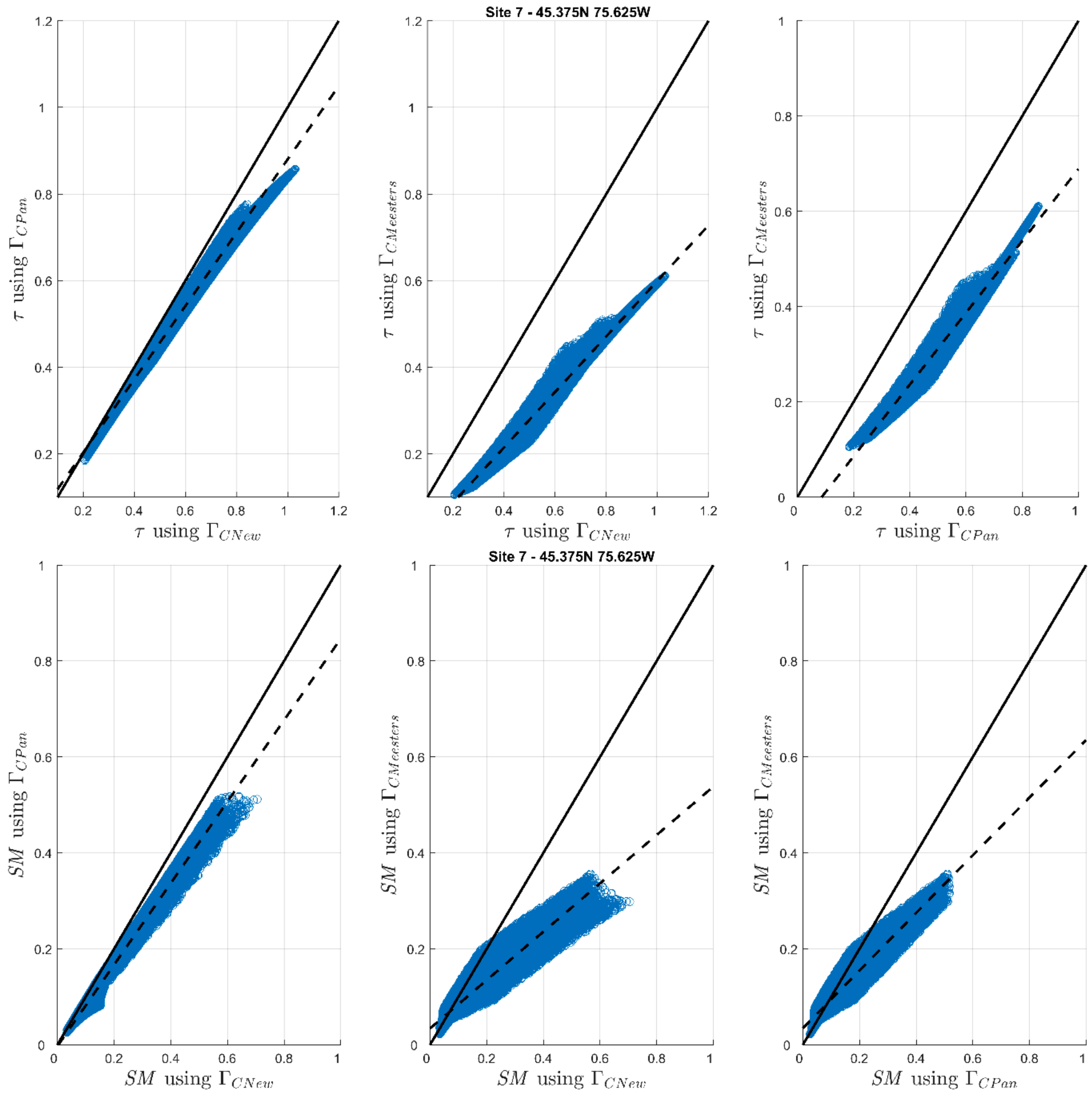

Figure S7. Same as Figure S2, but for Site 7.

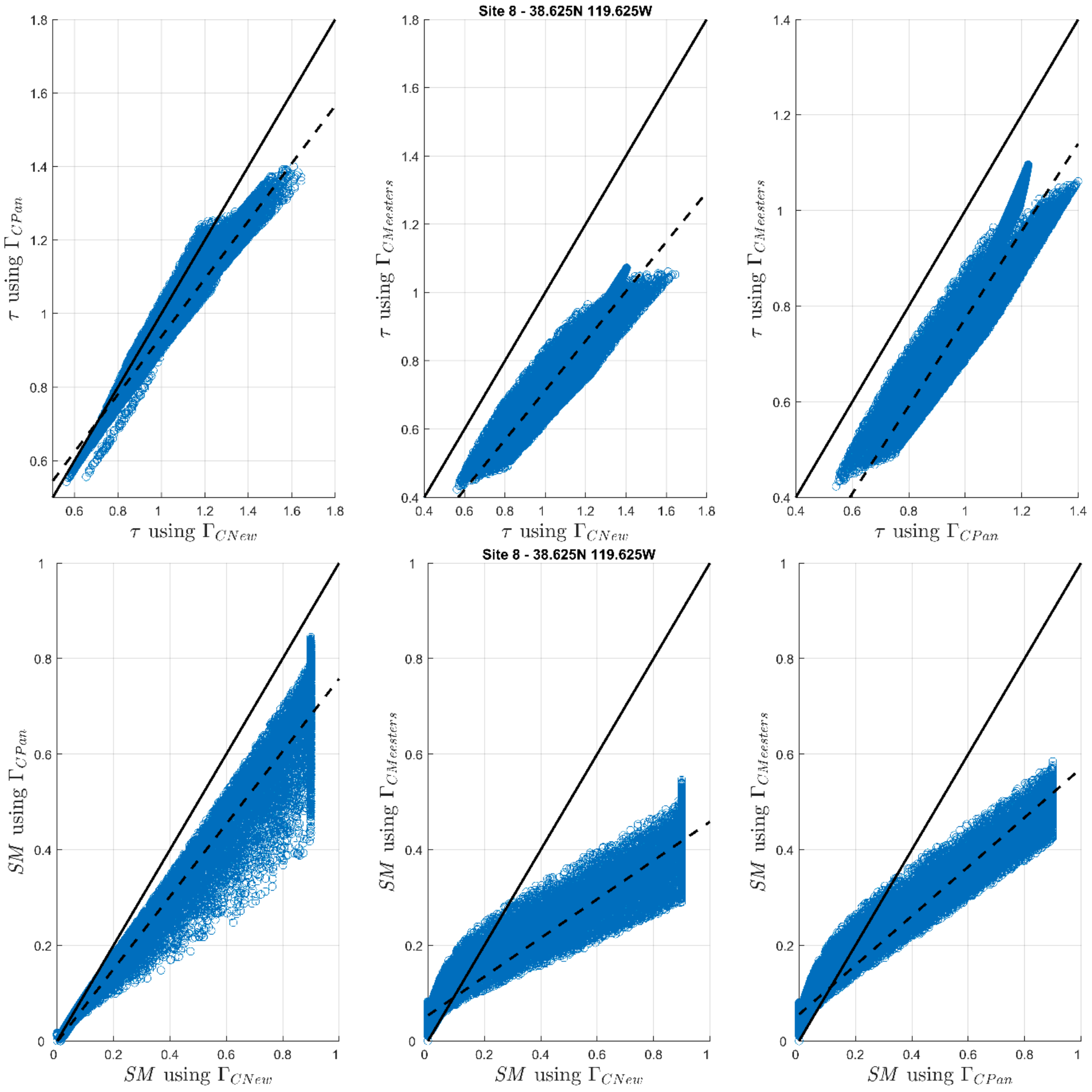

Figure S8. Same as Figure S2, but for Site 8.
